# Supplementary material for: Parental gender inequality and their children’s educational attainment, quality of life and mental health: An analysis from the Pelotas 1993 birth cohort in Brazil
Source: Glob Ment Health (Camb). 2026 Jan 26;13:e21. doi: 10.1017/gmh.2026.10139 (PMC12902877; doi:10.1017/gmh.2026.10139)
Supplement: Crossley et al. supplementary material [file S2054425126101393sup001.docx]

Supplementary Information for:

**Parental gender inequality and their children’s educational attainment, quality of life, and mental health: an analysis from the Pelotas 1993 birth cohort in Brazil.**

NA Crossley, L Czepielewski, AMB Menezes, F Wehrmeister, C Gama.

**CONTENT**

- **STROBE checklist**
- **Supplementary Figures**
  - **Figure S1:** Association between years of education and Couple’s Gender Inequality Index according to sex and family income.
  - **Figure S2:** Associations between outcomes and the individual domains of the Couple’s Gender Inequality Index.
  - **Figure S3:** Associations between outcomes and maternal or paternal disadvantage in the Education domain.
- **Supplementary Tables**
  - **Table S1:** Characteristics of participants born in 1993 in Pelotaas not included in this study.
  - **Table S2:** Stepwise models looking at years of education and Couple’s Gender Inequality Index.
  - **Table S3:** Stepwise models looking at quality of life and Couple’s Gender Inequality Index.
  - **Table S4:** Stepwise models looking at risk of depression at 18 years old and Couple’s Gender Inequality Index.
  - **Table S5:** Stepwise models looking at SDQ Emotional score at 15 years old and Couple’s Gender Inequality Index.

# STROBE Statement—checklist of items that should be included in reports of observational studies

|  | Item No. | | | Recommendation | Page  No. | | |
| --- | --- | --- | --- | --- | --- | --- | --- |
| **Title and abstract** | 1 | | | (*a*) Indicate the study’s design with a commonly used term in the title or the abstract | 1-3 | | |
|  |  |  |  | (*b*) Provide in the abstract an informative and balanced summary of what was done and what was found | 2-3 | | |
| Introduction | | | | | | | |
| Background/rationale | 2 | | | Explain the scientific background and rationale for the investigation being reported | 4-6 | | |
| Objectives | 3 | | | State specific objectives, including any prespecified hypotheses | 5-6 | | |
| Methods | | | | | | | |
| Study design | 4 | | | Present key elements of study design early in the paper | 6-10 | | |
| Setting | 5 | | | Describe the setting, locations, and relevant dates, including periods of recruitment, exposure, follow-up, and data collection | 6-7 | | |
| Participants | 6 | | | (*a*) *Cohort study*—Give the eligibility criteria, and the sources and methods of selection of participants. Describe methods of follow-up  *Case-control study*—Give the eligibility criteria, and the sources and methods of case ascertainment and control selection. Give the rationale for the choice of cases and controls  *Cross-sectional study*—Give the eligibility criteria, and the sources and methods of selection of participants | 10 | | |
|  |  |  |  | (*b*) *Cohort study*—For matched studies, give matching criteria and number of exposed and unexposed  *Case-control study*—For matched studies, give matching criteria and the number of controls per case |  | | |
| Variables | 7 | | | Clearly define all outcomes, exposures, predictors, potential confounders, and effect modifiers. Give diagnostic criteria, if applicable | 6-10 | | |
| Data sources/ measurement | 8* | | | For each variable of interest, give sources of data and details of methods of assessment (measurement). Describe comparability of assessment methods if there is more than one group | 6-10 | | |
| Bias | 9 | | | Describe any efforts to address potential sources of bias | 9-10 | | |
| Study size | 10 | | | Explain how the study size was arrived at | 6 | | |
| Quantitative variables | | 11 | Explain how quantitative variables were handled in the analyses. If applicable, describe which groupings were chosen and why | | 7-10 | | |
| Statistical methods | | 12 | (*a*) Describe all statistical methods, including those used to control for confounding | | 6-10 | | |
|  |  |  | (*b*) Describe any methods used to examine subgroups and interactions | | 6-10 | | |
|  |  |  | (*c*) Explain how missing data were addressed | |  | | |
|  |  |  | (*d*) *Cohort study*—If applicable, explain how loss to follow-up was addressed  *Case-control study*—If applicable, explain how matching of cases and controls was addressed  *Cross-sectional study*—If applicable, describe analytical methods taking account of sampling strategy | |  | | |
|  |  |  | (*e*) Describe any sensitivity analyses | | 9-10 | | |
| Results | | | | | | |  |
| Participants | | 13* | (a) Report numbers of individuals at each stage of study—eg numbers potentially eligible, examined for eligibility, confirmed eligible, included in the study, completing follow-up, and analysed | | 11 |  |  |
|  |  |  | (b) Give reasons for non-participation at each stage | |  |  |  |
|  |  |  | (c) Consider use of a flow diagram | |  |  |  |
| Descriptive data | | 14* | (a) Give characteristics of study participants (eg demographic, clinical, social) and information on exposures and potential confounders | |  |  |  |
|  |  |  | (b) Indicate number of participants with missing data for each variable of interest | |  |  |  |
|  |  |  | (c) *Cohort study*—Summarise follow-up time (eg, average and total amount) | | 7 |  |  |
| Outcome data | | 15* | *Cohort study*—Report numbers of outcome events or summary measures over time | | 11-15 |  |  |
|  |  |  | *Case-control study—*Report numbers in each exposure category, or summary measures of exposure | |  |  |  |
|  |  |  | *Cross-sectional study—*Report numbers of outcome events or summary measures | |  |  |  |
| Main results | | 16 | (*a*) Give unadjusted estimates and, if applicable, confounder-adjusted estimates and their precision (eg, 95% confidence interval). Make clear which confounders were adjusted for and why they were included | | 11-15 |  |  |
|  |  |  | (*b*) Report category boundaries when continuous variables were categorized | |  |  |  |
|  |  |  | (*c*) If relevant, consider translating estimates of relative risk into absolute risk for a meaningful time period | |  |  |  |
| Other analyses | | 17 | Report other analyses done—eg analyses of subgroups and interactions, and sensitivity analyses | | SI |  |  |
| Discussion | | | | | | |  |
| Key results | | 18 | Summarise key results with reference to study objectives | | 18 |  |  |
| Limitations | | 19 | Discuss limitations of the study, taking into account sources of potential bias or imprecision. Discuss both direction and magnitude of any potential bias | | 20-21 |  |  |
| Interpretation | | 20 | Give a cautious overall interpretation of results considering objectives, limitations, multiplicity of analyses, results from similar studies, and other relevant evidence | | 18-20 |  |  |
| Generalisability | | 21 | Discuss the generalisability (external validity) of the study results | | 20-21 |  |  |
| Other information | | |  | | | |  |
| Funding | | 22 | Give the source of funding and the role of the funders for the present study and, if applicable, for the original study on which the present article is based | | 21 |  |  |

*Give information separately for cases and controls in case-control studies and, if applicable, for exposed and unexposed groups in cohort and cross-sectional studies.

**Figure S1.**

***Figure S1. Association between Couple’s Gender Inequality and years of education according to a) Sex and b) Family Income.*** *Values are shown without correction for other confounders (sex, family income, gestational age, birth weight, non-white parent).*

**Figure S2.**

***Figure S2. Associations between outcomes and the individual domains of the Couple’s Gender Inequality Index.*** *Values are shown without correction for other confounders (sex, family income, gestational age, birth weight, non-white parent). Note the overall concordance with the combined index, indicating that all domains contribute to the associations observed with the composite measure.*

**Figure S3**

***Figure S3. Associations between outcomes and maternal or paternal disadvantage in the Education domain.*** *The top row shows results for Q1 (mothers have less education than fathers) and Q2 (mothers have equal or higher education), while the bottom row shows results for Q1 (fathers have less education than mothers) and Q2 (fathers have equal or higher education).* *Values are shown without correction for other confounders (sex, family income, gestational age, birth weight, non-white parent). Note that conditions of educational disadvantage in fathers did not show clear associations with child developmental outcomes.*

**Table S1. Characteristics of participants born in 1993 in Pelotas not included in this study.**

|  |  | **INCLUDED** | **NOT INCLUDED** | **Available data** | **P-Value** | **Test** |  |
| --- | --- | --- | --- | --- | --- | --- | --- |
| **Numbers** | N | 2852 | 2397 |  |  |  |  |
|  | (%) | 54.33% | 45.67% |  |  |  |  |
| **Sex** | % female | 50.70% | 50.25% | 99.96% | P=0.65 | X-squared 0.2 | df 1 |
| **Perinatal** | Good (≥8 visits) | 56.60% | 45.39% |  | P<0.0001 | X-squared 64.82 | df 1 |
| **Care** | Bad (<4 visits) | 7.60% | 16.26% | 99.50% | P<0.0001 | X-squared 94.37 | df 1 |
| **Adolescent** | <14yo | 1.02% | 1.60% |  | P=0.09 | X-squared 2.88 | df 1 |
| **Pregnancy** | 14-19yo | 29.24% | 44.32% | 88.57% | P<0.0001 | X-squared 11.97 | df 1 |
| **Maternal Education** [IQR] | | 7 [5 to 10] | 6 [4 to 8] | 99.70% | P<0.0001 | Wilcoxon W 3.9 x 10^^6^ | |
| **Family Income** (min salaries [IQR]) | | 2.9 [1.6 to 5.0] | 2.5 [1.5 to 4.5] | 96.66% | P<0.0001 | Wilcoxon W 3.5 x 10^^6^ | |

*IQR= interquartile range.*

**Table S2. Stepwise models looking at years of education and Couple’s Gender Inequality Index.**

| Years of education | exp (betas) | exp (SE) | P-value | exp (betas) | exp (SE) | P-value | exp (betas) | exp (SE) | P-value | exp (betas) | exp (SE) | P-value | exp (betas) | exp (SE) | P-value | exp (betas) | exp (SE) | P-value | exp (betas) | exp (SE) | P-value | exp (betas) | exp (SE) | P-value | exp (betas) | exp (SE) | P-value |
| --- | --- | --- | --- | --- | --- | --- | --- | --- | --- | --- | --- | --- | --- | --- | --- | --- | --- | --- | --- | --- | --- | --- | --- | --- | --- | --- | --- |
| (Intercept) | 7.676 | 1.016 | <0.0001 | 7.571 | 1.020 | <0.0001 | 7.369 | 1.020 | <0.0001 | 7.616 | 1.021 | <0.0001 | 5.891 | 1.204 | <0.0001 | 5.654 | 1.204 | <0.0001 | 6.413 | 1.211 | <0.0001 | 6.209 | 1.211 | <0.0001 | 7.191 | 1.023 | <0.0001 |
| SEX | 1.096 | 1.013 | <0.0001 | 1.126 | 1.028 | <0.0001 | 1.131 | 1.028 | <0.0001 | 1.140 | 1.028 | <0.0001 | 1.139 | 1.029 | <0.0001 | 1.145 | 1.029 | <0.0001 | 1.150 | 1.029 | <0.0001 | 1.154 | 1.029 | <0.0001 | 1.136 | 1.028 | <0.0001 |
| CGII-2nd quart. | 1.100 | 1.019 | <0.0001 | 1.102 | 1.029 | 0.0006 | 1.089 | 1.029 | 0.0025 | 1.088 | 1.029 | 0.0030 | 1.087 | 1.029 | 0.0032 | 1.087 | 1.029 | 0.0033 | 1.085 | 1.029 | 0.0039 | 1.102 | 1.033 | 0.0027 | 1.106 | 1.033 | 0.0017 |
| CGII-3rd quart. | 1.125 | 1.019 | <0.0001 | 1.146 | 1.028 | <0.0001 | 1.127 | 1.028 | <0.0001 | 1.1272 | 1.028 | <0.0001 | 1.121 | 1.028 | <0.0001 | 1.128 | 1.028 | <0.0001 | 1.128 | 1.028 | <0.0001 | 1.147 | 1.032 | <0.0001 | 1.147 | 1.032 | <0.0001 |
| CGII-4th quart. | 1.193 | 1.019 | <0.0001 | 1.233 | 1.028 | <0.0001 | 1.192 | 1.028 | <0.0001 | 1.189 | 1.028 | <0.0001 | 1.188 | 1.028 | <0.0001 | 1.208 | 1.028 | <0.0001 | 1.204 | 1.028 | <0.0001 | 1.245 | 1.032 | <0.0001 | 1.243 | 1.032 | <0.0001 |
| SEX:CGII-2nd quart. | NA | NA | NA | 0.995 | 1.039 | 0.9011 | 0.996 | 1.040 | 0.9073 | 0.989 | 1.040 | 0.7784 | 0.991 | 1.040 | 0.8201 | 0.990 | 1.040 | 0.7875 | 0.991 | 1.040 | 0.8189 | 0.988 | 1.040 | 0.7620 | 0.993 | 1.040 | 0.8583 |
| SEX:CGII-3rd quart. | NA | NA | NA | 0.965 | 1.039 | 0.3419 | 0.962 | 1.039 | 0.3106 | 0.958 | 1.039 | 0.2615 | 0.964 | 1.039 | 0.3393 | 0.964 | 1.039 | 0.3328 | 0.964 | 1.039 | 0.3351 | 0.960 | 1.039 | 0.2885 | 0.959 | 1.039 | 0.2665 |
| SEX:CGII-4th quart. | NA | NA | NA | 0.940 | 1.039 | 0.0999 | 0.930 | 1.039 | 0.0556 | 0.927 | 1.039 | 0.0448 | 0.930 | 1.039 | 0.0552 | 0.926 | 1.039 | 0.0438 | 0.925 | 1.039 | 0.0415 | 0.923 | 1.039 | 0.0362 | 0.928 | 1.039 | 0.0482 |
| Family Income | NA | NA | NA | NA | NA | NA | 1.009 | 1.001 | <0.0001 | 1.008 | 1.001 | <0.0001 | 1.008 | 1.001 | <0.0001 | 1.008 | 1.001 | <0.0001 | 1.008 | 1.001 | <0.0001 | 1.014 | 1.003 | 0.0001 | 1.016 | 1.003 | <0.0001 |
| Parental racial identification | NA | NA | NA | NA | NA | NA | NA | NA | NA | 0.879 | 1.016 | <0.0001 | 0.882 | 1.017 | <0.0001 | 0.890 | 1.017 | <0.0001 | 0.893 | 1.017 | <0.0001 | 0.894 | 1.017 | <0.0001 | NA | NA | NA |
| Gestational age | NA | NA | NA | NA | NA | NA | NA | NA | NA | NA | NA | NA | 1.007 | 1.005 | 0.1667 | 1.006 | 1.005 | 0.1922 | 1.000 | 1.005 | 0.9186 | 1.000 | 1.005 | 0.9840 | NA | NA | NA |
| Living with both parents | NA | NA | NA | NA | NA | NA | NA | NA | NA | NA | NA | NA | NA | NA | NA | 1.083 | 1.014 | <0.0001 | 1.082 | 1.014 | <0.0001 | 1.082 | 1.014 | <0.0001 | NA | NA | NA |
| Birth Weight | NA | NA | NA | NA | NA | NA | NA | NA | NA | NA | NA | NA | NA | NA | NA | NA | NA | NA | 1.000 | 1.000 | 0.0042 | 1.000 | 1.000 | 0.0058 | NA | NA | NA |
| CGII-2nd quart.: Family income | NA | NA | NA | NA | NA | NA | NA | NA | NA | NA | NA | NA | NA | NA | NA | NA | NA | NA | NA | NA | NA | 0.995 | 1.004 | 0.2017 | 0.994 | 1.004 | 0.1540 |
| CGII-3rd quart.: Family income | NA | NA | NA | NA | NA | NA | NA | NA | NA | NA | NA | NA | NA | NA | NA | NA | NA | NA | NA | NA | NA | 0.994 | 1.004 | 0.1518 | 0.994 | 1.004 | 0.1018 |
| CGII-4th quart.: Family income | NA | NA | NA | NA | NA | NA | NA | NA | NA | NA | NA | NA | NA | NA | NA | NA | NA | NA | NA | NA | NA | 0.992 | 1.004 | 0.0281 | 0.990 | 1.004 | 0.0051 |

*CGII= Couple’s Gender Inequality Index.*

**Table S3. Stepwise models looking at quality of life and Couple’s Gender Inequality Index.**

| Quality of Life | betas | SE | P-value | betas | SE | P-value | betas | SE | P-value | betas | SE | P-value | betas | SE | P-value | betas | SE | P-value | betas | SE | P-value | betas | SE | P-value | betas | SE | P-value |
| --- | --- | --- | --- | --- | --- | --- | --- | --- | --- | --- | --- | --- | --- | --- | --- | --- | --- | --- | --- | --- | --- | --- | --- | --- | --- | --- | --- |
| (Intercept) | 283.357 | 1.544 | <0.0001 | 283.818 | 1.937 | <0.0001 | 280.760 | 1.942 | <0.0001 | 283.692 | 1.976 | <0.0001 | 268.478 | 18.912 | <0.0001 | 265.365 | 18.847 | <0.0001 | 274.687 | 19.352 | <0.0001 | 275.289 | 19.423 | <0.0001 | 280.592 | 2.249 | <0.0001 |
| SEX | -9.291 | 1.384 | <0.0001 | -10.244 | 2.785 | 0.0002 | -9.875 | 2.753 | 0.0003 | -9.245 | 2.732 | 0.0007 | -9.113 | 2.750 | 0.0009 | -8.630 | 2.741 | 0.0017 | -8.309 | 2.744 | 0.0025 | -8.412 | 2.751 | 0.0023 | -9.852 | 2.758 | 0.0004 |
| CGII-2nd quart. | 7.576 | 1.971 | 0.0001 | 7.890 | 2.804 | 0.0049 | 6.579 | 2.775 | 0.0178 | 6.472 | 2.752 | 0.0188 | 6.879 | 2.763 | 0.0128 | 6.875 | 2.751 | 0.0125 | 6.749 | 2.751 | 0.0142 | 6.628 | 3.204 | 0.0387 | 6.744 | 3.220 | 0.0363 |
| CGII-3rd quart. | 6.531 | 1.941 | 0.0008 | 5.417 | 2.765 | 0.0502 | 3.477 | 2.739 | 0.2044 | 3.504 | 2.716 | 0.1971 | 3.561 | 2.734 | 0.1928 | 4.089 | 2.725 | 0.1336 | 4.139 | 2.726 | 0.1290 | 3.054 | 3.142 | 0.3312 | 2.851 | 3.155 | 0.3663 |
| CGII-4th quart. | 9.987 | 1.970 | <0.0001 | 8.940 | 2.799 | 0.0014 | 5.304 | 2.798 | 0.0582 | 5.188 | 2.775 | 0.0617 | 5.443 | 2.789 | 0.0511 | 6.808 | 2.793 | 0.0149 | 6.583 | 2.793 | 0.0185 | 5.555 | 3.221 | 0.0847 | 6.193 | 3.208 | 0.0537 |
| SEX:CGII-2nd quart. | NA | NA | NA | -0.518 | 3.945 | 0.8956 | -0.872 | 3.909 | 0.8234 | -1.415 | 3.878 | 0.7152 | -1.805 | 3.902 | 0.6436 | -1.976 | 3.886 | 0.6111 | -1.826 | 3.885 | 0.6384 | -1.814 | 3.896 | 0.6416 | -0.895 | 3.918 | 0.8194 |
| SEX:CGII-3rd quart. | NA | NA | NA | 2.186 | 3.886 | 0.5739 | 2.431 | 3.846 | 0.5274 | 2.062 | 3.815 | 0.5889 | 1.929 | 3.838 | 0.6153 | 1.868 | 3.822 | 0.6250 | 1.805 | 3.823 | 0.6369 | 1.941 | 3.830 | 0.6123 | 2.476 | 3.852 | 0.5205 |
| SEX:CGII-4th quart. | NA | NA | NA | 2.071 | 3.943 | 0.5995 | 0.974 | 3.899 | 0.8028 | 0.649 | 3.867 | 0.8666 | 0.799 | 3.890 | 0.8373 | 0.485 | 3.875 | 0.9003 | 0.413 | 3.873 | 0.9150 | 0.489 | 3.879 | 0.8996 | 1.013 | 3.903 | 0.7953 |
| Family Income | NA | NA | NA | NA | NA | NA | 1.030 | 0.113 | <0.0001 | 0.926 | 0.113 | <0.0001 | 1.005 | 0.118 | <0.0001 | 0.965 | 0.118 | <0.0001 | 0.957 | 0.118 | <0.0001 | 0.721 | 0.403 | 0.0736 | 1.087 | 0.398 | 0.0063 |
| Parental racial identification | NA | NA | NA | NA | NA | NA | NA | NA | NA | -10.747 | 1.612 | <0.0001 | -10.433 | 1.617 | <0.0001 | -9.704 | 1.618 | <0.0001 | -9.427 | 1.621 | <0.0001 | -9.456 | 1.622 | <0.0001 | NA | NA | NA |
| Gestational age | NA | NA | NA | NA | NA | NA | NA | NA | NA | NA | NA | NA | 0.376 | 0.487 | 0.4393 | 0.339 | 0.485 | 0.4838 | -0.172 | 0.539 | 0.7498 | -0.173 | 0.539 | 0.7486 | NA | NA | NA |
| Living with both parents | NA | NA | NA | NA | NA | NA | NA | NA | NA | NA | NA | NA | NA | NA | NA | 6.612 | 1.411 | <0.0001 | 6.512 | 1.411 | <0.0001 | 6.517 | 1.412 | <0.0001 | NA | NA | NA |
| Birth weight | NA | NA | NA | NA | NA | NA | NA | NA | NA | NA | NA | NA | NA | NA | NA | NA | NA | NA | 0.003 | 0.001 | 0.0308 | 0.003 | 0.001 | 0.0288 | NA | NA | NA |
| CGII-2nd quart.:Family income | NA | NA | NA | NA | NA | NA | NA | NA | NA | NA | NA | NA | NA | NA | NA | NA | NA | NA | NA | NA | NA | 0.099 | 0.483 | 0.8367 | -0.056 | 0.480 | 0.9074 |
| CGII-3rd quart.: Family income | NA | NA | NA | NA | NA | NA | NA | NA | NA | NA | NA | NA | NA | NA | NA | NA | NA | NA | NA | NA | NA | 0.316 | 0.455 | 0.4876 | 0.106 | 0.452 | 0.8140 |
| CGII-4th quart.:Family income | NA | NA | NA | NA | NA | NA | NA | NA | NA | NA | NA | NA | NA | NA | NA | NA | NA | NA | NA | NA | NA | 0.286 | 0.439 | 0.5147 | -0.165 | 0.431 | 0.7024 |

*CGII= Couple’s Gender Inequality Index.*

**Table S4. Stepwise models looking at risk of depression at 18 years old and Couple’s Gender Inequality Index.**

| Risk of Depression | exp (betas) | exp (SE) | P-value | exp (betas) | exp (SE) | P-value | exp (betas) | exp (SE) | P-value | exp (betas) | exp (SE) | P-value | exp (betas) | exp (SE) | P-value | exp (betas) | exp (SE) | P-value | exp (betas) | exp (SE) | P-value | exp (betas) | exp (SE) | P-value | exp (betas) | exp (SE) | P-value |
| --- | --- | --- | --- | --- | --- | --- | --- | --- | --- | --- | --- | --- | --- | --- | --- | --- | --- | --- | --- | --- | --- | --- | --- | --- | --- | --- | --- |
| (Intercept) | 0.0394 | 1.2286 | <0.0001 | 0.0579 | 1.2743 | <0.0001 | 0.0797 | 1.2922 | <0.0001 | 0.0681 | 1.3048 | <0.0001 | 0.0879 | 9.7717 | 0.2860 | 0.1075 | 9.9102 | 0.3308 | 0.035 | 10.512 | 0.1547 | 0.035 | 10.627 | 0.1561 | 0.0838 | 1.3888 | <0.0001 |
| SEX | 2.9596 | 1.2092 | <0.0001 | 1.6426 | 1.3705 | 0.1153 | 1.6184 | 1.3713 | 0.1273 | 1.5740 | 1.3722 | 0.1517 | 1.5776 | 1.3732 | 0.1506 | 1.5136 | 1.3746 | 0.1927 | 1.466 | 1.376 | 0.2303 | 1.467 | 1.376 | 0.2298 | 1.6166 | 1.3715 | 0.1283 |
| CGII-2nd quart. | 0.7999 | 1.2581 | 0.3310 | 0.4072 | 1.5726 | 0.0472 | 0.4466 | 1.5741 | 0.0756 | 0.4470 | 1.5746 | 0.0761 | 0.4500 | 1.5749 | 0.0787 | 0.4457 | 1.5758 | 0.0756 | 0.454 | 1.576 | 0.0828 | 0.398 | 1.717 | 0.0883 | 0.3670 | 1.7184 | 0.0640 |
| CGII-3rd quart. | 0.8552 | 1.2493 | 0.4822 | 0.4444 | 1.5410 | 0.0607 | 0.5180 | 1.5438 | 0.1298 | 0.5120 | 1.5443 | 0.1235 | 0.5158 | 1.5445 | 0.1277 | 0.4854 | 1.5460 | 0.0972 | 0.489 | 1.546 | 0.1006 | 0.416 | 1.668 | 0.0870 | 0.4151 | 1.6828 | 0.0912 |
| CGII-4th quart. | 0.5823 | 1.2830 | 0.0300 | 0.3490 | 1.6133 | 0.0278 | 0.4807 | 1.6222 | 0.1299 | 0.4762 | 1.6227 | 0.1254 | 0.4810 | 1.6231 | 0.1307 | 0.4259 | 1.6265 | 0.0793 | 0.434 | 1.627 | 0.0869 | 0.615 | 1.810 | 0.4121 | 0.6739 | 1.8144 | 0.5077 |
| SEX:CGII-2nd quart. | NA | NA | NA | 2.6253 | 1.6992 | 0.0687 | 2.6023 | 1.7027 | 0.0723 | 2.6609 | 1.7038 | 0.0663 | 2.5699 | 1.7060 | 0.0772 | 2.6293 | 1.7078 | 0.0709 | 2.546 | 1.709 | 0.0812 | 2.544 | 1.709 | 0.0813 | 2.5933 | 1.7023 | 0.0733 |
| SEX:CGII-3rd quart. | NA | NA | NA | 2.5566 | 1.6638 | 0.0652 | 2.4105 | 1.6685 | 0.0857 | 2.4639 | 1.6697 | 0.0786 | 2.4544 | 1.6707 | 0.0802 | 2.4989 | 1.6724 | 0.0749 | 2.494 | 1.673 | 0.0757 | 2.494 | 1.672 | 0.0756 | 2.4291 | 1.6683 | 0.0829 |
| SEX:CGII-4th quart. | NA | NA | NA | 2.1317 | 1.7576 | 0.1795 | 2.2665 | 1.7608 | 0.1481 | 2.2964 | 1.7618 | 0.1421 | 2.2891 | 1.7623 | 0.1439 | 2.3295 | 1.7638 | 0.1362 | 2.342 | 1.765 | 0.1341 | 2.426 | 1.768 | 0.1200 | 2.3666 | 1.7643 | 0.1292 |
| Family Income | NA | NA | NA | NA | NA | NA | 0.8782 | 1.0387 | 0.0006 | 0.8887 | 1.0384 | 0.0017 | 0.8857 | 1.0393 | 0.0016 | 0.8951 | 1.0384 | 0.0032 | 0.899 | 1.038 | 0.0043 | 0.893 | 1.105 | 0.2578 | 0.8579 | 1.1105 | 0.1437 |
| Parental racial identification | NA | NA | NA | NA | NA | NA | NA | NA | NA | 1.5580 | 1.1978 | 0.0140 | 1.5576 | 1.1982 | 0.0142 | 1.4710 | 1.1999 | 0.0342 | 1.428 | 1.201 | 0.0516 | 1.428 | 1.201 | 0.0516 | NA | NA | NA |
| Gestational age | NA | NA | NA | NA | NA | NA | NA | NA | NA | NA | NA | NA | 0.9938 | 1.0603 | 0.9158 | 0.9969 | 1.0607 | 0.9586 | 1.059 | 1.069 | 0.3918 | 1.059 | 1.069 | 0.3916 | NA | NA | NA |
| Living with both parents | NA | NA | NA | NA | NA | NA | NA | NA | NA | NA | NA | NA | NA | NA | NA | 0.5852 | 1.1917 | 0.0023 | 0.586 | 1.192 | 0.0024 | 0.590 | 1.192 | 0.0027 | NA | NA | NA |
| Birth Weight | NA | NA | NA | NA | NA | NA | NA | NA | NA | NA | NA | NA | NA | NA | NA | NA | NA | NA | 1.000 | 1.000 | 0.0524 | 1.000 | 1.000 | 0.0548 | NA | NA | NA |
| CGII-2nd quart.:Family income | NA | NA | NA | NA | NA | NA | NA | NA | NA | NA | NA | NA | NA | NA | NA | NA | NA | NA | NA | NA | NA | 1.051 | 1.129 | 0.6853 | 1.0797 | 1.1329 | 0.5392 |
| CGII-3rd quart.:Family income | NA | NA | NA | NA | NA | NA | NA | NA | NA | NA | NA | NA | NA | NA | NA | NA | NA | NA | NA | NA | NA | 1.056 | 1.119 | 0.6277 | 1.0807 | 1.1257 | 0.5120 |
| CGII-4th quart.:Family income | NA | NA | NA | NA | NA | NA | NA | NA | NA | NA | NA | NA | NA | NA | NA | NA | NA | NA | NA | NA | NA | 0.909 | 1.139 | 0.4652 | 0.9164 | 1.1452 | 0.5199 |

*CGII= Couple’s Gender Inequality Index.*

**Table S5. Stepwise models looking at SDQ Emotional scores at 15 years old and Couple’s Gender Inequality Index.**

| SDQ emotional | exp (betas) | exp (SE) | P-value | exp (betas) | exp (SE) | P-value | exp (betas) | exp (SE) | P-value | exp (betas) | exp (SE) | P-value | exp (betas) | exp (SE) | P-value | exp (betas) | exp (SE) | P-value | exp (betas) | exp (SE) | P-value | exp (betas) | exp (SE) | P-value | exp (betas) | exp (SE) | P-value |
| --- | --- | --- | --- | --- | --- | --- | --- | --- | --- | --- | --- | --- | --- | --- | --- | --- | --- | --- | --- | --- | --- | --- | --- | --- | --- | --- | --- |
| (Intercept) | 3.880 | 1.022 | <0.0001 | 3.704 | 1.028 | <0.0001 | 3.888 | 1.029 | <0.0001 | 3.704 | 1.030 | <0.0001 | 4.149 | 1.321 | <0.0001 | 4.316 | 1.321 | <0.0001 | 3.825 | 1.331 | <0.0001 | 3.643 | 1.332 | 0.0000 | 3.777 | 1.034 | <0.0001 |
| SEX | 1.042 | 1.020 | 0.0373 | 1.142 | 1.039 | 0.0005 | 1.124 | 1.039 | 0.0026 | 1.118 | 1.039 | 0.0040 | 1.121 | 1.040 | 0.0033 | 1.115 | 1.040 | 0.0052 | 1.112 | 1.040 | 0.0066 | 1.119 | 1.040 | 0.0041 | 1.129 | 1.040 | 0.0018 |
| CGII-2nd quart. | 0.949 | 1.028 | 0.0585 | 1.053 | 1.040 | 0.1874 | 1.061 | 1.040 | 0.1344 | 1.067 | 1.040 | 0.1004 | 1.067 | 1.041 | 0.1038 | 1.066 | 1.041 | 0.1103 | 1.068 | 1.041 | 0.0974 | 1.151 | 1.049 | 0.0033 | 1.145 | 1.049 | 0.0042 |
| CGII-3rd quart. | 0.901 | 1.028 | 0.0001 | 0.948 | 1.041 | 0.1822 | 0.969 | 1.041 | 0.4284 | 0.971 | 1.041 | 0.4615 | 0.970 | 1.042 | 0.4593 | 0.963 | 1.042 | 0.3567 | 0.964 | 1.042 | 0.3698 | 0.995 | 1.049 | 0.9130 | 0.996 | 1.048 | 0.9375 |
| CGII-4th quart. | 0.897 | 1.029 | 0.0001 | 0.928 | 1.042 | 0.0670 | 0.971 | 1.043 | 0.4764 | 0.975 | 1.043 | 0.5480 | 0.984 | 1.043 | 0.6938 | 0.967 | 1.043 | 0.4258 | 0.970 | 1.043 | 0.4764 | 1.003 | 1.050 | 0.9440 | 0.986 | 1.050 | 0.7760 |
| SEX:CGII-2nd quart. | NA | NA | NA | 0.815 | 1.057 | 0.0002 | 0.828 | 1.057 | 0.0007 | 0.830 | 1.057 | 0.0008 | 0.831 | 1.058 | 0.0010 | 0.835 | 1.058 | 0.0013 | 0.833 | 1.058 | 0.0011 | 0.826 | 1.058 | 0.0007 | 0.823 | 1.057 | 0.0005 |
| SEX:CGII-3rd quart. | NA | NA | NA | 0.905 | 1.057 | 0.0704 | 0.919 | 1.057 | 0.1298 | 0.921 | 1.057 | 0.1393 | 0.917 | 1.058 | 0.1223 | 0.919 | 1.058 | 0.1332 | 0.918 | 1.058 | 0.1262 | 0.912 | 1.058 | 0.1017 | 0.915 | 1.057 | 0.1116 |
| SEX:CGII-4th quart. | NA | NA | NA | 0.936 | 1.058 | 0.2391 | 0.961 | 1.058 | 0.4863 | 0.959 | 1.058 | 0.4598 | 0.954 | 1.059 | 0.4123 | 0.960 | 1.059 | 0.4726 | 0.960 | 1.059 | 0.4703 | 0.953 | 1.059 | 0.4036 | 0.957 | 1.059 | 0.4344 |
| Family Income | NA | NA | NA | NA | NA | NA | 0.985 | 1.002 | <0.0001 | 0.987 | 1.002 | <0.0001 | 0.986 | 1.002 | <0.0001 | 0.986 | 1.002 | <0.0001 | 0.987 | 1.002 | <0.0001 | 0.998 | 1.006 | 0.7959 | 0.994 | 1.006 | 0.3383 |
| Parental racial identification | NA | NA | NA | NA | NA | NA | NA | NA | NA | 1.169 | 1.023 | <0.0001 | 1.167 | 1.023 | <0.0001 | 1.157 | 1.023 | <0.0001 | 1.153 | 1.023 | <0.0001 | 1.154 | 1.023 | 0.0000 | NA | NA | NA |
| Gestational age | NA | NA | NA | NA | NA | NA | NA | NA | NA | NA | NA | NA | 0.997 | 1.007 | 0.6792 | 0.998 | 1.007 | 0.7311 | 1.004 | 1.008 | 0.6171 | 1.004 | 1.008 | 0.5893 | NA | NA | NA |
| Living with both parents | NA | NA | NA | NA | NA | NA | NA | NA | NA | NA | NA | NA | NA | NA | NA | 0.908 | 1.021 | <0.0001 | 0.909 | 1.021 | 0.0000 | 0.909 | 1.021 | 0.0000 | NA | NA | NA |
| Birth Weight | NA | NA | NA | NA | NA | NA | NA | NA | NA | NA | NA | NA | NA | NA | NA | NA | NA | NA | 1.000 | 1.000 | 0.0725 | 1.000 | 1.000 | 0.0738 | NA | NA | NA |
| CGII-2nd quart.:Family income | NA | NA | NA | NA | NA | NA | NA | NA | NA | NA | NA | NA | NA | NA | NA | NA | NA | NA | NA | NA | NA | 0.977 | 1.008 | 0.0045 | 0.977 | 1.008 | 0.0041 |
| CGII-3rd quart.:Family income | NA | NA | NA | NA | NA | NA | NA | NA | NA | NA | NA | NA | NA | NA | NA | NA | NA | NA | NA | NA | NA | 0.989 | 1.007 | 0.1203 | 0.990 | 1.007 | 0.1741 |
| CGII-4th quart.:Family income | NA | NA | NA | NA | NA | NA | NA | NA | NA | NA | NA | NA | NA | NA | NA | NA | NA | NA | NA | NA | NA | 0.988 | 1.007 | 0.0820 | 0.992 | 1.007 | 0.2461 |

*CGII= Couple’s Gender Inequality Index.*
